# Skin Lesion Detection Algorithms in Whole Body Images

**DOI:** 10.3390/s21196639

**Published:** 2021-10-06

**Authors:** Michał H. Strzelecki, Maria Strąkowska, Michał Kozłowski, Tomasz Urbańczyk, Dorota Wielowieyska-Szybińska, Marcin Kociołek

**Affiliations:** 1 Institute of Electronics, Lodz University of Technology, Żeromskiego 116, 90-924 Łódź, Poland; maria.strakowska@p.lodz.pl (M.S.); michal.kozlowski@uwm.edu.pl (M.K.); marcin.kociolek@p.lodz.pl (M.K.); 2 Department of Mechatronics and Technical and IT Education, Faculty of Technical Science, University of Warmia and Mazury, 11-041 Olsztyn, Poland; 3 Skopia Estetic Clinic, Josepha Conrada 79, 31-357 Kraków, Poland; turbanczyk@poczta.dmt.com.pl (T.U.); dwielow@gmail.com (D.W.-S.); 4 Smoluchowski Institute of Physics, Jagiellonian University, Łojasiewicza 11, 30-348 Kraków, Poland

**Keywords:** skin lesion detection, whole body system, algorithm fusion

## Abstract

Melanoma is one of the most lethal and rapidly growing cancers, causing many deaths each year. This cancer can be treated effectively if it is detected quickly. For this reason, many algorithms and systems have been developed to support automatic or semiautomatic detection of neoplastic skin lesions based on the analysis of optical images of individual moles. Recently, full-body systems have gained attention because they enable the analysis of the patient’s entire body based on a set of photos. This paper presents a prototype of such a system, focusing mainly on assessing the effectiveness of algorithms developed for the detection and segmentation of lesions. Three detection algorithms (and their fusion) were analyzed, one implementing deep learning methods and two classic approaches, using local brightness distribution and a correlation method. For fusion of algorithms, detection sensitivity = 0.95 and precision = 0.94 were obtained. Moreover, the values of the selected geometric parameters of segmented lesions were calculated and compared for all algorithms. The obtained results showed a high accuracy of the evaluated parameters (error of area estimation <10%), especially for lesions with dimensions greater than 3 mm, which are the most suspected of being neoplastic lesions.

## 1. Introduction

Melanoma is a malignant skin tumor and is considered the deadliest of skin cancers. The reason for this is its rapid growth and ease of metastasis. In 2018, more than 100,000 people died of skin cancer. Although in developed countries the number of deaths from this type of cancer is decreasing (in the USA there has been an average decrease in death rate in years 2013–2017 by 6.3%), the number of cases is still growing (e.g., in Poland there has been an increase from 1600 cases in 1999 to 3600 in 2018). Melanoma is almost 100% curable if detected quickly. For this reason, it is very important to increase preventive measures aimed at promoting behaviors that prevent the formation of this cancer and awareness of the importance of screening. It is very important to popularize mobile applications that allow independent tests to be conducted of a suspected naevi detected on the body. Such an analysis, carried out based on a photo taken with a smartphone, allows obtaining information about the potential risk of detecting a neoplastic lesion, which must of course be verified by a dermatologist. Mobile applications developed for skin cancer detection recently gained popularity; the thorough analysis provided in [1] reports 43 such applications. However, only two of these are both currently available and were analyzed and evaluated in scientific publications. These are SkinVision [2] and SpotMole [3]. The first implements conditional generative adversarial neural network segmentation and SVM for mole classification, resulting in sensitivity in the range of 57–72% and specificity of 27–50% [4]. The second application employs the ABCD rule, providing sensitivity and specificity equal to 43% and 80%, respectively [4]. Such parameters show that reliability of verified mobile applications, despite significant development skin lesion detection and classification algorithms, is still far from clinical standards. However, mobile phones are implemented as a part of teledermatological systems that are now becoming a reality. The comparison of four different teledermatological workflows reported in [5] demonstrated that teledermatology is efficient in diagnosis, reaching a sensitivity greater than 90% and a specificity greater than 80%. One of the limitations of such systems is the image acquisition device that defines the quality of the captured lesion images: a system equipped with a dermoscope outperformed these based on smartphones by achieving a greater probability of correct cancer detection and lower probability of unnecessary biopsy [5].

Other approaches implemented for melanoma detection are full-body (or whole-, total-body) systems (FBS). Contrary to classic investigations, where the attention is focused on a particular lesion and a single image is taken using a dermoscope or a high-quality camera, FBS captures a number of photographs of the whole body. Such a system allows not only lesion detection, but also the possibility of comparing images acquired at different times. In this manner, it is possible to detect lesions that evolve in time and to find new lesions that appeared since the previous examination. Both types of changes are suspected to be malicious. The objective of this paper is to present and compare three algorithms aimed at detection and further segmentation of moles in whole body optical images. Such algorithms will be a part of a future FBS that allows fast scanning of a patient’s body, and detection and registration of lesions, because some parts of the body overlap in a number of acquired images. This FBS would be intended as a device of dermatology clinics enabling fast and accurate analysis of the whole body to detect suspicious lesions. Due to its fast examination, the system could be implemented for screening or epidemiological studies.

FBSs are not very common in the literature. The vast majority of research focuses on detection and classification of single lesion images. There are a plethora of different approaches developed for this task—good surveys regarding recent advancements in melanoma detection can be found in [6,7]. Among various techniques one should pay attention to deep neural networks, a tool widely used in different biomedical image analysis tasks. Efficiency of DNNs in dermoscopic image segmentation and analysis was also previously demonstrated—recent applications of various deep learning systems for skin diagnosis are presented in [8,9,10,11]. An FBS for detection of new melanocytic lesions is described in [12]. It implements 3D stereo capture system with 22 acquisition units and LoG filtering for lesion segmentation, resulting in 90% recall. Another FBS is presented in [13,14]. It is based on a cabin equipped with 21 high resolution cameras that capture the patient’s body in 24 turnable positions. In [15], wide body images are analyzed to detect suspicious pigmented lesions. Larger lesions (diameter > 3 mm) were manually segmented, and ABCD features were then calculated for each lesion. The logistic regression classifier provided over 84% sensitivity and 75% accuracy in detection of suspicious lesions. An ACNN-based approach to detection and classification of suspicious lesions in whole body images is presented in [16]. The blob detection algorithm was implemented for lesion identification, whereas various CNN architectures were applied for classification of detected lesions into six classes (including nonsuspicious and suspicious lesions). The presented system was tested on a large number of lesions (over 33,000) and reached 80% average accuracy for suspicious classification endpoints. The developed CNN algorithm also obtained good agreement with dermatologists’ assessment. None of the above systems was tested in a dermatological clinic.

In our system we implemented two novel lesion detection and segmentation algorithms. In contrast to previously implemented solutions, based on global image filtering [12,17], these algorithms make use of local lesion properties, related to their gray-level distribution and shape, that allow lesion discrimination from the skin.

- The first approach is based on local skin modelling and analysis of histograms of selected color components. When an analyzed image fragment contains a lesion, it is reflected in its histogram. Analysis of histogram shapes enable efficient lesion segmentation (Section 2.2.1).
- The second algorithm assumes that the vast majority of lesions present in the whole-body image can be modelled by a finite number of lesion patterns. Two-dimensional cross-correlation functions are calculated for such patterns and the analyzed image. Functions’ maxima indicate the lesion position in the image. Detected lesions’ regions are further segmented by the active contour approach to detect the lesion shape (Section 2.2.2).

Another novelty of this paper lies in an efficiency comparison of the above algorithms that implement classic image processing approaches with a deep network model (YOLOv3) adopted for analysis of whole-body skin images (Section 2.2.3). Moreover, a fusion of these three algorithms was performed to check if a combination of two different object detection paradigms (the classic one, based on the selection of predetermined object features such as its shape and intensity, and deep learning-based approach, where object features are determined by the network internally) gives better results than either of them separately.

## 2. Materials and Methods

### 2.1. Image Acquisition System

**Figure 1:**
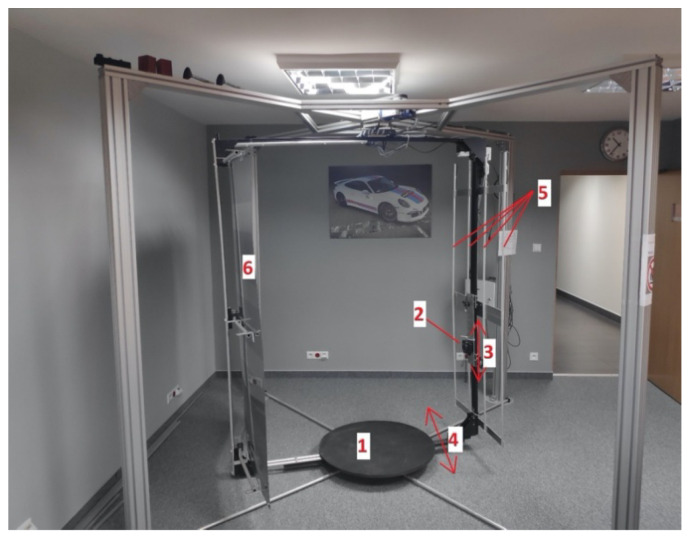
The device used for capturing pictures of whole area of human skin. 1—The motionless platform on which the photographed person stands; 2—the Sony DSC RX100 digital camera; 3—illustration of camera movement in vertical direction; 4—illustration of camera movement around the photographed person; 5—LED strips providing strong and homogenous illumination; 6—gray background.

The constructed device is presented in Figure 1. To capture the pictures of the whole area of the skin, the device is equipped with a system which automatically changes position of an RGB digital camera in two directions: vertically and around the person being photographed (see 3 and 4 in Figure 1). As an imaging device in the constructed system, a commercially available digital camera (SONY DSC RX100) was used (see 2 in Figure 1). This digital camera is equipped with a 20 MP (5488 × 3664 pixels) back illuminated CMOS sensor and a lens with focal length in the range of 8.8–25.7 mm and up to 2.9× optical zoom. During examination the patient stands on a motionless platform at the center of the device (see 1 in Figure 1). The boom to which the camera is attached gradually rotates and stops at a selected number (usually 8) of equidistant angular positions. At each angular position the camera is moved vertically and stops at a selected number of positions (usually 5 equidistant positions: ranging from 35 to 165 cm above the level of platform at the center of device). For each stop of the camera in its vertical movement the camera captures a picture. Usually, the examination contains 32 overlapping pictures which cover the whole area of skin of the photographed person; however, the number of photos can be easily modified by changing the parameters of examination. The construction of the device allows modification of the distance between the camera and the axis of rotation of the boom (in range from 60 to 120 cm); however, in most conducted examinations the optimal 80 cm distance between the camera lens and rotation axis was used. To provide sufficiently strong and homogeneous illumination of the photographed person, a system of four vertical LED strips with diffusers is used. The LED strips are attached to the rotating boom on both sides of the camera (see 5 in Figure 1). The color temperature of the LEDs is 4000 K (so-called neutral-white [18]) and the total luminous power of the systems is over 8000 lumens. The choice of these parameters was rather arbitrary and resulted from the experiments performed. The chosen color temperature was also best perceived by the patients. The device is also equipped with a gray background (see 6 in Figure 1), which rotates with the camera boom. Due to this set-up, for each position of the camera the background is situated behind the person being photographed. To reduce the total examination time, the device can be equipped with up to four additional digital cameras. In that case the number of stops in vertical movement is significantly reduced.

### 2.2. Skin Image Analysis

Using the acquisition system described in Section 2.1, 480 skin images for 15 patients were collected, of which the 50 with the highest number of lesions were selected for further analysis. These images represent different fragments of the human body. All these images were preprocessed to detect the skin region only. Different color spaces were tested to find the optimum separation of the body from the background; finally, it was decided to use RGB space and modified skin detection algorithm described in [19]. Next, a hair-removal algorithm was applied, based on the dull razor approach [20]. Preprocessed images underwent segmentation with use of three algorithms, as described in the following sections.

#### 2.2.1. Local Skin Model Based Approach

**Figure 2:**
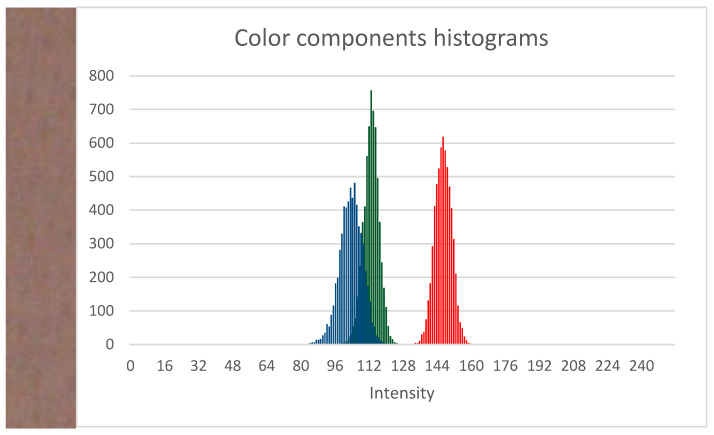
Example of healthy skin fragments with corresponding brightness histograms for the three color components—red, green, and blue.

Overall, human skin is not homogeneous. It has various structures that are to a greater or lesser extent visible in partial human body images acquired in visible light. Such structures may include subcutaneous blood vessels, pathological and benign melanocytic lesions, hemangiomas, wounds, scars, discolorations, hair follicles, hairs, anatomical structures such as the nipples and the navel, and many others. Some of these structures are more or less visible depending on the parameters and properties of the image acquisition system, such as resolution, type and brightness of lighting, sensitivity of optical sensor, focal length, and lens aperture. Despite the heterogeneity, small fragments of healthy skin are characterized by clustered near-Gaussian intensity histograms of the three primary colors (Figure 2).

**Figure 3:**
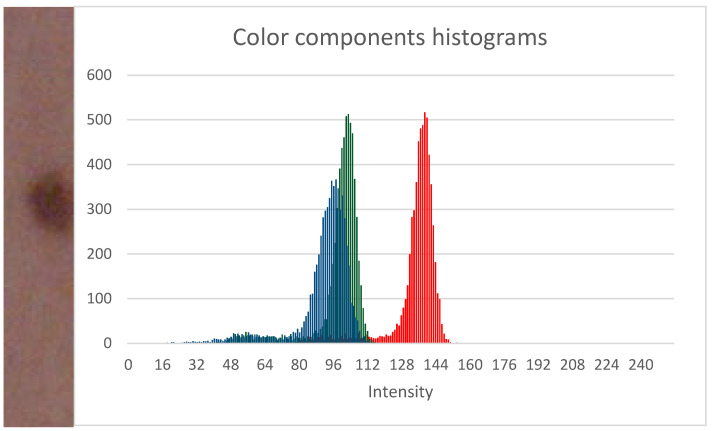
Example of a skin fragment with a melanocytic change with corresponding brightness histograms for the three color components—red, green, and blue.

The above property is used by the melanocytic lesion detection algorithm we propose. Analyzing the histograms of skin fragments with melanocytic nevi, we found that this type of nevus causes the formation of a kind of “tail” on the side of low brightness values, especially in the histograms for the green and blue components (Figure 3). Identification of pixels belonging to the “tails” for green and blue components leads to melanocytic lesion discovery.

To identify intensities belonging to the tail, one should identify histogram distribution for the healthy skin. This is possible if the nevus is a smaller part of the analyzed fragment of the image and the remaining part is healthy skin. In such a situation most of the histograms resemble a Gaussian distribution. Because the “tail” is present only for the lower intensity side, the border between normal skin and the lesion intensities can be estimated as the mode (highest point) of histogram, minus the width of the Gaussian distribution, which in turn can be estimated as the maximum histogram value minus the mode.

**Figure 4:**
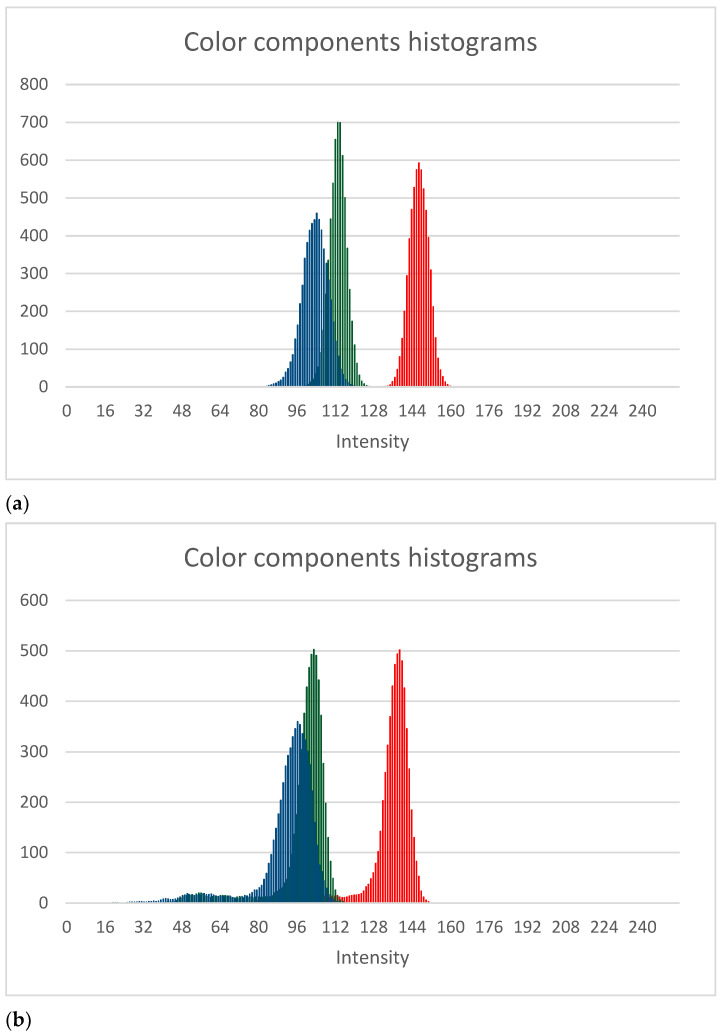
Smoothed version of histograms: (**a**) a healthy skin fragment (from Figure 2) (**b**) skin fragments with a melanocytic change (from Figure 3).

To improve mode estimation histogram smoothing, an averaging filter can be applied (see Figure 4). The performed experiments demonstrated that such filtering reduces the relative error in determining the area of the nevus by a few percent.

The lesion detection procedure is described below as Algorithm 1. This algorithm must be applied to overlapping fragments all over the patient’s body. Pixels identified as background, underwear, or hair are excluded from the analysis.
**Algorithm 1** Segmentation method based on local skin model **Function** GetLesionMaskSkinModel   **Data:**  J_SKIN_—input RGB image   **Result:**
*LM*—lesion mask   Divide input image into overlapping tiles: J_Tile_ ← J_SKIN_   Create empty lesion mask *LM* having the size of J_SKIN_   **foreach** J_Tile_ in J_SKIN_      Extract green and blue channels from tile: *G*, *B* ← J_Tile_ Determine a histogram for the green and blue color components. Smooth the histograms with the averaging filter. Find mode and maximum value for both histograms. Find the threshold values for the green and blue components              $\begin{array}{c} th_{G}=mode_{G}-\;\left(max_{G}-mode_{G}\right)\\ th_{B}=mode_{B}-\;\left(max_{B}-mode_{B}\right) \end{array}$      Find the lesion mask           $LM\left(x,y\right)\;=\;\left\{\begin{array}{cc} 1 & for\;\left(G\left(x,y\right)\;<\;th_{G}\right)\&\left(B\left(x,y\right)\;<\;th_{B}\right)\\ 0 & otherwise \end{array}\right.$   **end**   Label lesion mask in the way that each separate lesion will have a unique numerical identifier ** End**

#### 2.2.2. Correlation-Based Approach

This method assumes that there are a finite number of mole patterns that dominate in the whole-body images. Thus, proper identification of such patterns allows detection of the majority of skin moles based, e.g., on a correlation method. Masks that contain most typical mole patterns (selected based on analysis on acquired images) move across the image to estimate a set of correlation functions. The normalized cross-correlation between the image and the template is estimated by the formula:

(1) $$\gamma \left(u,v\right)\;=\sum_{x,y}\frac{\left[\;f\left(x,y\right)\;-\;{\bar{f}}_{u,v}\;\right]\left[\;t\left(x-u,y-v\right)\;-\;\bar{t}\;\right]}{{\left\{\;\sum_{x,y}{\left[\;f\left(x,y\right)\;-\;{\bar{f}}_{u,v}\;\right]}^{2}\sum_{x,y}{\left[\;t\left(x-u,y-v\right]\;-\;\bar{t}\;\right]}^{2}\;\right\}}^{0.5}}$$

where

*f(x,y)*—is the image,
*t(x,y)*—is the lesion template,
$\bar{t}—$ is the mean of template,
${\bar{f}}_{u,v}$—is the mean of *f(x,y)* in the region under the template.

Next, spots are detected in the coordinates of the correlation matrix where it reaches local maxima. A set of discriminated spots is a combination of partial results separately obtained for correlation with each mask.

**Figure 5:**
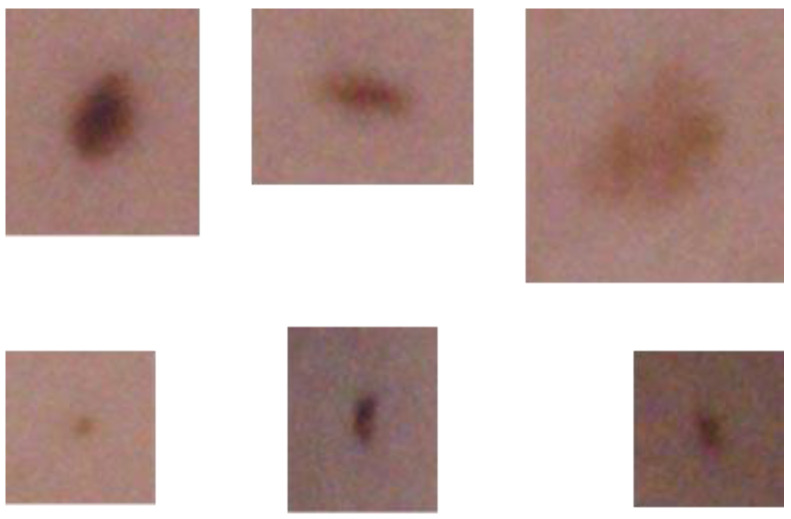
Exemplary masks with the spots.

These spots that contribute to the pattern set were validated by a medical doctor as “for inspection”. The choice of the proper spot masks is the essential factor of the developed algorithm. Due to this, several masks with the patient’s spots were extracted from the images and the cross-correlation was calculated. The masks that lead to the high value of correlation maxima, and to a large number of maxima should be considered as mole patterns. Such masks are characterized with the biggest similarity to the greatest number of other spots. Thus, they are good candidates for finding similar types of spots in other images (including those that are not a part of currently analyzed set). An exemplary set of masks is shown in the Figure 5.

**Figure 6:**
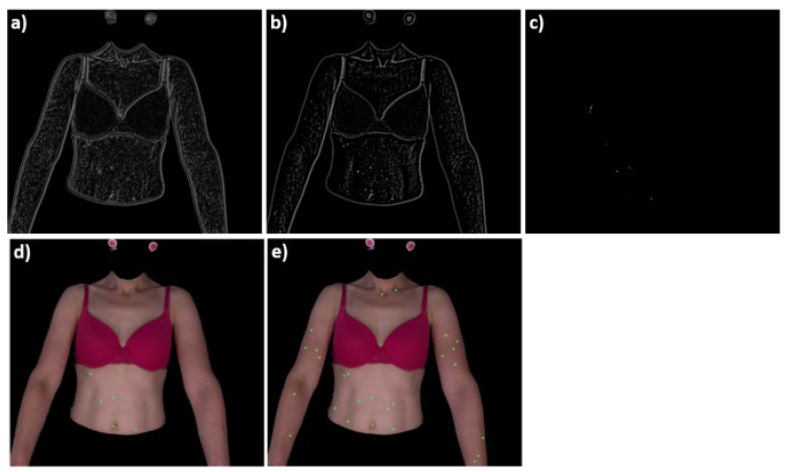
Subsequent steps of spot detection (one mask analysis). (**a**) correlation matrix, (**b**) suppressed local maxima’s below threshold, (**c**) artefacts removed, (**d**) spots detected for given mask,(**e**) combined results for all masks.

The correlation matrix is generated for every mask and the processed image. Searching the spots is based on finding the local maxima values in the correlation matrix (shown in Figure 6a). A few steps must be performed to delete unnecessary artefacts (Figure 6b). First, the areas where the correlation is less than zero are deleted (Figure 6c). Second, a binary image is created by thresholding the correlation matrix. Finally, searching for spots that are bigger than 1 mm and smaller (or equal) than the biggest mask image takes place. The rest are rejected (Figure 6d). The combination of all spots detected by each mask generates the final image with detected spots, as shown in Figure 6e.

**Figure 7:**
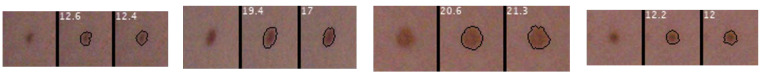
Exemplary masks 1 and its segmentation 2—by dermatologist, 3—by the software.

Finally, detected spots are segmented using the Active Contour (AC) method [21] using the Y channel of YCbCr color space. Centers of the masks are the starting points for the AC function for region expansion. Sample segmentation results are presented in Figure 7. The method’s operation is summarized by Algorithm 2.
**Algorithm 2** Spot detection based on cross-correlation between mask and the image.**Function** spotDetectionCCorrelation   **Data:**  J_SKIN_—input RGB image      MS—list of model spots (masks)   **Result:** FDS—detected spots (location, area, MaxFeretDiameter)   Selecting green channel from images: J_G_ ← J_SKIN_, MS_G_ ← MS   **foreach** ms_i_ in MS_G_      Cross-correlation calculation: cc_i_ = crossCorrelation(J_G_, ms_i_)      Spots detection: ds_i_ = maximaDetection(cc_i_, sensit)      Adding detected spots to the list: DS = DS + ds_i_   **end**   Removing duplicated spots: DS = removeDuplications(DS,minDist)   Removing spots close to background: DS = removeCloseToBG(DS,J_G_)   Detected spots segmentation: FDS = spotsSegmentation(DS) **end**

#### 2.2.3. Deep Learning-Based Approach

Another approach we propose is based on the method of object detection (indicating their place in an image with their height and width) using the YOLO model [22]. This is an object detector that uses functions learned by the deep convolutional neural network to detect an object. Improved versions have since been released, including the one we used in this approach. YOLOv3 uses several modifications to improve training and increase performance, including better skeleton classifier and multi-scale predictions, which are fully described in [23]. This model is characterized by extremely fast operation, and can even be used in devices that process images in real time, especially if it uses the computing power of the GPU (graphics processing unit).

**Figure 8:**
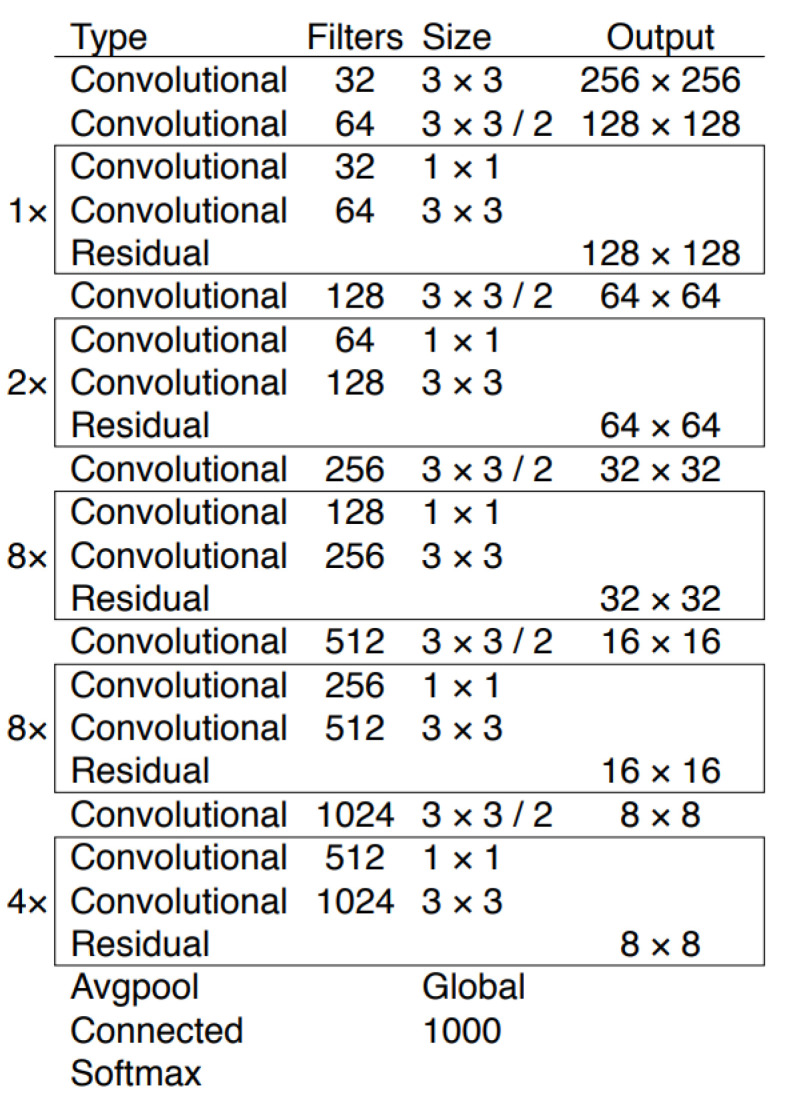
Architecture of YOLOv3 [24].

In this study, the YOLOv3 model was implemented based on the Darknet [24] framework. This model makes use of only convolutional layers, making it a fully convolutional network (FCN). It has 53 convolutional layers with skip connections and upsampling layers (see Figure 8 for details).

Minor modifications were made to adapt the architecture to the problem under study. The main element was the limitation of decision classes. We are looking for skin lesions, and the rest of the image should be ignored (background), so the class is limited to one. The input layer size was 416 × 416 pixels. The usual approach is to reduce the dimensions of the original image to the size of the input layer, but then we would lose information about small skin lesions.

**Figure 9:**
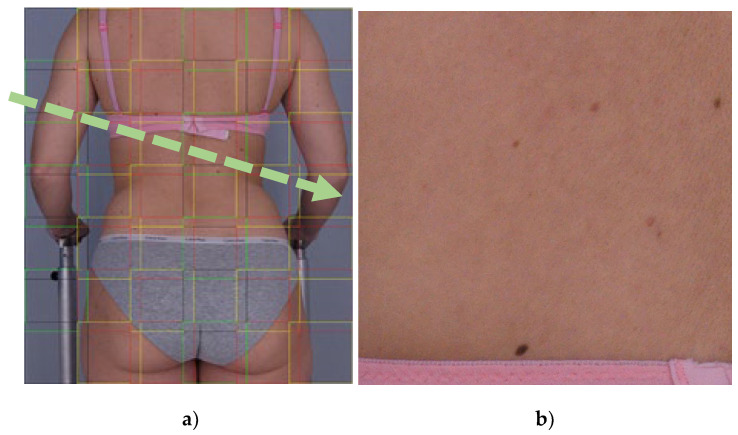
Framed input image (**a**), image frame transferred to the input of the CNN model (**b**).

Therefore, we decided to cut the original image into frames that overlap (as shown in Figure 9) to avoid frames with border object location. The mean width of the overlapping was 118 pixels.

The obtained frames are characterized by small (compared to the background area) regions of interest. That is why only frames with skin lesions were used in the process of training the network. In total, for 32 analyzed images, 945 frames with lesions were obtained. They were divided into training (67% frames) and validation (33% frames) sets. To increase the number of training samples, data augmentation was applied. This was undertaken by adding 8 different copies of the frame (4 × flip, 3 × rotate and transpose). To improve the network accuracy, during the learning process, the saturation and exposure were changed randomly by up to 20% [25]. Network validation was performed using three-fold cross-validation to assess its generalization. The division of frames into the test and training set was made in such a manner that the overlapping areas of the frames did not duplicate in both sets. Network performance parameters were calculated as mean values of these obtained for each of the three validation schemes.

**Figure 10:**
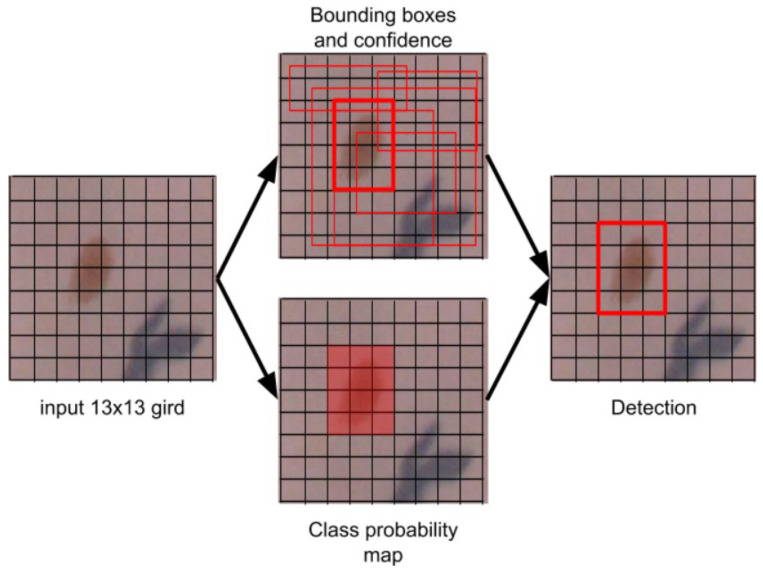
Simple representation of how “you look only once” (Yolo) deep network detects the location of the skin lesion.

Neural network training was performed using the Darknet framework, using an NVIDIA GF RTX 2080 Ti GPU with 11 GB VRAM and 4352 CUDA cores. To reduce the possibility of overfitting the data, a transfer learning technique was used: each convolutional layer was initiated from the Darknet-53 model pre-trained in ImageNet, a dataset containing 1.5 million natural images of various origins in 1000 classes [26]. The training was completed at 20,000 iterations. The rest of the parameter values were set provided in the DarkNet specification [24]. As in the case of training, during testing the full resolution image is divided into frames. Each frame is divided into regions, for which the network estimates a probability that the given region contains a lesion (see Figure 10).

**Figure 11:**
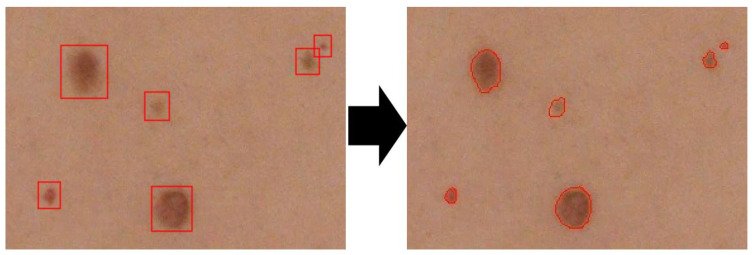
Segmentation of detected skin lesions. Segmented lesions are marked with lines.

The boundary box containing the highest probability values represents a model decision that locates the object of interest. Next, the center point, dimensions along the vertical and horizontal axes, and the confidence prediction of the lesion were calculated. Finally, all detected lesions were marked in the full resolution image. The problem of duplicate detections appearing due to overlapping image frames was solved by applying the Non-Maximum Suppression (NMS) method [27]. Next, the skin lesion segmentation was performed within each previously detected bounding box. For this purpose, the Otsu method [28,29] was used preceded by median filtration (kernel size equal to 5 pixels) to reduce the noise in the box. The method’s operation is summarized by Algorithm 3. Sample segmentation results are presented in Figure 11.
**Algorithm 3** Spot detection based on CNN**Function** spotDetectionCNN   **Data**:  J_SKIN_—input RGB image      M_W_—weights of the trained model      M_C_—net configuration   **Result**: FDS—detected spots (location, area, confidencePrediction)   Cut the original image into overlapping frames: J_FRAMES_ ← J_SKIN_   **foreach** j_FRAME_ **in** J_FRAMES_      Run network and gather predictions lists:       NET_OUT_ = netDetections(j_FRAME_, M_W,_ M_C_)      Add detection to list: NET += netOUT   **end**   Removing duplicated detections: NET = removeDuplicationsNMS(NET)   **foreach** net **in** NET      Detected spots segmentation: FDS = spotsSegmentation(net)   **end** **end** **where:** netDetections (j_FRAME_, M_W,_ M_C_)—the DNN model processes the image frame returning the detections in the form of a bounding box. removeDuplicationsNMS(NET)—Non Maximum Suppression method. spotsSegmentation(net)—processing areas of interest using the Otsu method and median filtering.

## 3. Results and Discussion

Lesion detection reliability was evaluated by comparing the obtained segmentation results for each method with ground truth images where lesions were marked by a dermatologist. Analysis was performed for three lesion size groups: 1–2 mm, 2–5 mm and >5 mm.

Summarized results obtained for all datasets are presented in Table 1. T (true) is the number of reference lesions marked manually by experienced dermatologist. TP (true positives) is the number of real lesions identified by the given method, FN (false negatives) is the number of lesions not recognized by the method and FP (false positives) is the number of detected image objects by the given method which do not represent lesions (FP represent, e.g., artifacts). The differences in the T numbers between the methods is caused by small differences in preprocessing stages between the methods, mainly in background and underwear detection. False positives were calculated only for all cases because the actual size of lesion were assessed based on annotated lesions.

Sensitivity and precision as measures of lesion detection quality are presented in Table 2. These measures are defined as follows:

(2) $$sensitivity=\frac{TruePositives}{TruePositives+FalseNegatives}$$

(3) $$precision=\frac{TruePositives}{TruePositives+FalsePositives}$$

Precision was calculated for all cases because the actual size of lesions was assessed based on annotated lesions and no false positives count was available for size classes.

Three lesion sizes (1–2 mm, 2–5 mm, >5 mm) were assumed for checking of detection accuracy for all these ranges. The reason for separately analyzing the biggest lesions (>5 mm) is that we wanted to check if their detection did not deteriorate for some cases. If this occurred, the developed methods would not correctly detect large nevi which are potential neoplastic changes. This table also presents results for an approach that considers a fusion of all methods. In these data, a lesion is considered as detected when is indicated by at least two methods. Resulting images constructed based on results of three segmentation methods (using “majority voting” rule) are compared with ground truth images and the same parameters are calculated to assess the combined method efficiency.

**Figure 12:**
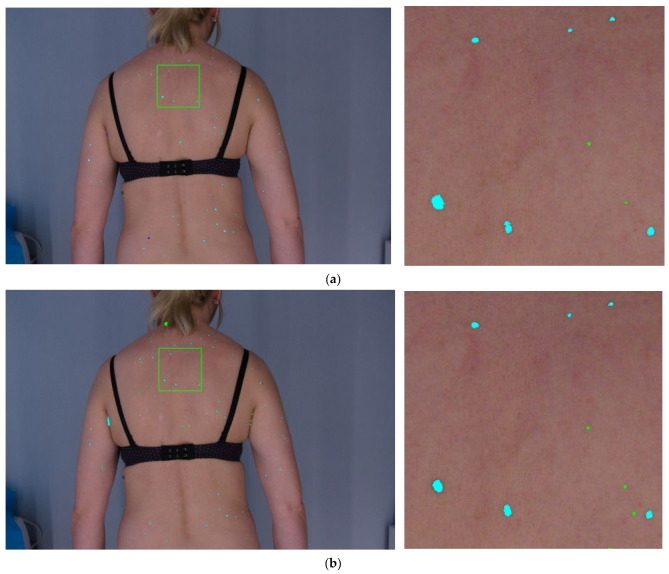

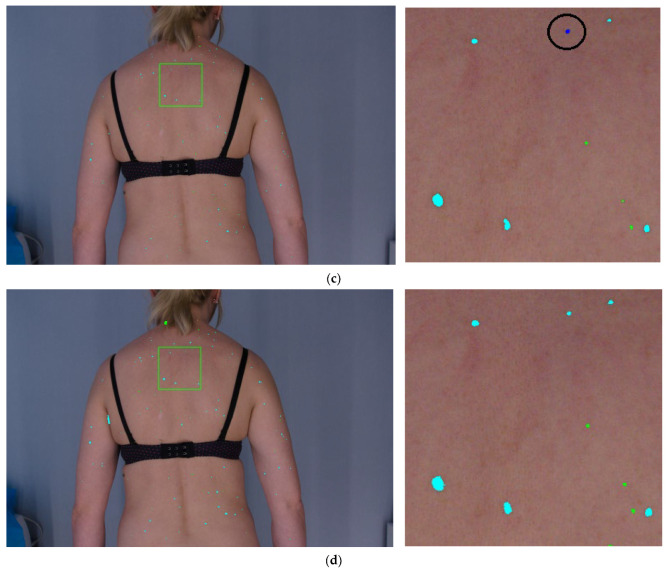
Sample lesion detection results (whole body and magnified fragment) by four methods: histogram (**a**), correlation (**b**), CNN (**c**) and fusion (**d**). Dark blue objects are lesions marked by dermatologist not detected by the algorithm (FN), green areas represent non-lesion objects identified by one of detection methods (FP), light blue objects represent the correctly detected lesions (TP). For the sake of clarification, an example of FN is additionally marked by a black circle on (**c**).

It is clearly seen from Table 1 that the best results in terms of precision value were obtained for the CNN-based approach. Even with a relatively small training dataset the network was able to minimize the number of FP cases, while also failing to detect a relatively small number of lesions (FN). Most errors generated by this approach were caused by untypical shadows located in specific body fragments (like in the auricle) which disturbed the appearance of the lesion. The histogram-based method represents the opposite behavior. It performs well in not missing true lesions. However, the price paid for this is high detection of objects that do not represent lesions at all. This was mainly caused by jewelry (worn by some patients), head hair and underwear segmentation problems, skin folds, men’s warts, and shadows in the image. The approach based on correlation resulted in precision compared to the other two methods, while the sensitivity was slightly lower. The reason for errors in this case was shadows in the image due to uneven lighting. This caused many false detections. This algorithm also failed when the lesion was placed near the edge of the patient’s figure or close to the underwear. The content of the mask is also very important. Masks must be characterized by a large gradient between the lesion and the background, otherwise, many areas where there is no lesion are detected. The masks must also have an even background. If not, shaded areas are detected which do not contain moles at all. Promising results were obtained for the “majority voting” method. This provided highest sensitivity whereas precision was smaller than given by the CNN. This was caused by the large amount of FP for both histogram and correlation-based approaches. Thus, the resulting number of FP after applying the merging rule was also high. Importantly, all methods worked well for larger lesions (size >2 mm), which may represent potential neoplastic changes. Segmentation results provided by the four methods are illustrated in Figure 12.

The obtained results were also compared to these reported in literature. Two full-body systems described in [12] were used as a reference. In the previous study, a sensitivity of 0.9 was achieved for lesions, with a precision of 0.5 in the case of small (3 mm) lesions and 0.8 for larger new lesions (5 and 7 mm). In our study we demonstrated higher sensitivity for all algorithms; the highest was obtained for the fusion method (0.96). Precision for all lesions in our case was lowest for the histogram-based approach, whereas it was significantly higher for CNN and fusion approaches (0.94 and 0.91, respectively). Thus a “majority voting” method outperformed both sensitivity and precision of the described system, justifying the correctness of the adopted segmentation methods and opening the way for further tests. In [17], a system for lesion detection in optical images of larger body fragments (arm, forearm) was presented. The detection algorithm used there is based on difference-of-Gaussian (DoG) filters followed by an SVM classifier. The reported results show sensitivity in the range of 0.80–0.85 and accuracy in the range of 0.76–0.79, depending on the SVM working conditions. A comparison of the results is shown in Table 3. For the second FBS [13,14], quality measures were provided for lesion matching procedures only. The accuracy of the detection of lesions was summarized by the statement that the system detects almost 100% of the skin moles, which, however, makes it impossible to compare the obtained results with our solution. The other systems described in [15,16] are oriented for detection and classification of suspicious lesions in whole-body images; thus, detection results presented there cannot be compared with ours.

In addition, the quality of the applied lesion segmentation algorithms was investigated. There were three such algorithms applied, a different one for each detection method. For the histogram method, segmentation of nevi was performed based on the analysis of local image brightness distributions. In the case of correlation, approach lesions were segmented using the active contour approach. In deep learning, detection of lesions was undertaken with the use of YOLO networks, whereas for their segmentation, the Otsu thresholding was used. To evaluate the accuracy of each segmentation technique, the selected geometrical parameters were calculated for detected skin lesions: area, diameter of equivalent circle (a circle that has the same area as the lesion), and three features of ellipse that approximates lesion’s shape—major and minor axes sizes, angle between major axis, and Ox axis. In this case, analysis was performed for two lesion size groups: 1–3 mm and >3 mm because according to clinical evidence most melanomas are bigger than 3 mm [30]. The reference values were the geometrical parameters calculated for the mole’s outlines marked by a dermatologist. A relative error (3) was determined for each of the parameters:

(4) $$\Delta P=\frac{\left(\left|P-P_{der}\right|\right)}{P_{der}}\cdot 100\%$$

where:

P—the measured value of the parameter for nevi detected by various methods,
$P_{der}$—the parameter value measured for the outlines of lesion marked by a dermatologist.

The obtained error results, averaged for 100 analyzed lesions, are presented in Table 4. The error values for the segmentation results constituting a fusion of three methods were also determined, i.e., the logical sum of the superimposed results of the lesion segmentation for individual methods was assumed as the final result of segmentation (Fusion rows in Table 4).

It should be noted that the geometric parameters are salient features taken into account when assessing individual nevi by a trained dermatologist. The diameter is especially important when comparing the images of the melanocytic nevus taken over a period of time [30,31,32]. A significant increase in the diameter of a nevus between consecutive examinations is a strong indicator that the melanocytic nevi can be potentially dangerous. Considering each method separately, the Active Contour provides the most accurate segmentation. This confirms the repeatedly proven effectiveness of this method in various tasks of detecting objects in images. This method provides the lowest average values of the relative measurement error of all analyzed geometric parameters of the nevi. Such an approach has proven to be particularly effective for small nevi with diameter <3 mm. The fusion method provides the best measurement accuracy for large lesions (>3 mm). This result shows that, in general, all segmentation methods tend to detect a slightly smaller lesion than is the ground truth. Taking into account the logical sum of all segmentation results eliminates this effect, ensuring relatively accurate measurement of the geometry of the lesions (errors do not exceed 10% for all calculated parameters). This also results in better lesion shape reproduction—the Dice coefficient achieved the biggest values (0.80 and 0.86 depending on lesion size) for the fusion approach. In general, these errors are smaller for large lesions than for small ones, which is understandable because small lesions (especially these about 1 mm in size) are not accurately reproduced in the image and their segmentation is problematic. However, from the point of view of detecting suspicious nevi, it is important to accurately measure the geometry of any lesion bigger than 3 mm due to the highest probability of developing neoplastic changes.

A significant limitation of our study is the reduced dataset used for network training. The reason for this is the need to develop analysis algorithms strictly for images recorded by the system under development (the number of imaged volunteers was limited due to the COVID-19 pandemic), because in the future it will be used in a dermatology clinic for screening. Therefore, we focused on images, with properties determined by the implemented cameras, lighting, and acquisition method. These elements influence algorithm performance, which may be different for images acquired in other conditions (e.g., those available in public databases).

## 4. Conclusions

In this study, three methods of segmentation of nevi in the patient’s full body images were presented along with their quantitative analysis. The preliminary results are promising, particularly for the bigger lesions (size >3 mm) where fusion methods that combine the results of the individual approaches ensure good both detection and segmentation results. The majority vote fusion exhibits the highest sensitivity among all three approaches. Further work will focus on testing and optimization of these methods, especially on extended datasets on new patients. Moreover, the effort will be made to reduce the number of FP detections to improve the system’s precision. We expect that, in particular, increasing the size of the training sets with new photos (annotated photos, which we are currently gathering in large quantity) will lead to an even better performance of the CNN. We also plan to develop and implement image matching methods to identify the same lesions that appear in different photos taken for different positions of the patient’s body. This will enable the development of complete documentation of all patient’s moles, determination of their geometric parameters, and their further analysis by a dermatologist.

## Figures and Tables

**Table 1:** Lesion counts for 3 segmentations methods and for results obtained by considering all method outcomes.

Size [mm]	Histogram				Correlation				CNN				Fusion (Majority Voting)			
	T	TP	FN	FP	T	TP	FN	FP	T	TP	FN	FP	T	TP	FN	FP
1–2	626	578	48		635	523	112		619	559	60		623	575	48	
2–5	725	705	20		728	704	24		715	700	15		725	712	13	
>5	25	24	1		25	24	1		25	24	1		25	24	1	
All	1376	1307	69	854	1388	1251	137	292	1359	1283	76	75	1373	1311	62	157

**Table 2:** Lesion detection quality measurements for 3 segmentation methods and for results obtained by considering all method outcomes.

Size [mm] (# Lesions)	Histogram		Correlation		CNN		Fusion (Majority Voting)	
	Sensitivity	Precision	Sensitivity	Precision	Sensitivity	Precision	Sensitivity	Precision
1–2 (628)	0.92		0.82		0.90		0.92	
2–5 (726)	0.97		0.97		0.98		0.98	
>5 (25)	0.96		0.96		0.96		0.96	
All (1379)	0.95	0.60	0.90	0.81	0.94	0.94	0.96	0.91

**Table 3:** Comparison of systems’ performance.

Reference	Size [mm]	Sensitivity	Precison
Bogo et al. [12]	1–3	0.90	0.50
	>5	0.90	0.80
Taeg et al. [17]	all	0.80–0.85	-
Ours (Fusion approach)	1–2	0.92	-
	>5	0.96	-
	all	0.96	0.91

**Table 4:** Averaged relative errors [%] for geometrical parameters calculated and Dice coefficient [unitless] for lesion characterization.

	Size [mm]	Area	Diameter of Equivalent Circle	Ellipse Major Axis	Ellipse Minor Axis	Ellipse Angle	Dice Coefficient
Histogram (brightness distribution)	1–3	24.62	12.49	0.54	14.36	16.18	0.79
	>3	14.80	8.19	0.45	8.74	10.69	0.86
Correlation (Active contour)	1–3	20.14	10.89	0.56	12.91	14.42	0.74
	>3	23.79	13.54	0.35	12.55	14.12	0.81
CNN (Otsu threshold.)	1–3	28.53	15.99	0.47	18.27	19.65	0.77
	>3	21.72	12.46	0.51	13.09	13.17	0.79
Fusion (majority voting)	1–3	24.35	15.41	0.56	16.76	24.28	0.80
	>3	9.84	4.95	0.41	7.33	8.37	0.86

## Data Availability

The data presented in this study are not publicly available because optical full-body images of patients constitute sensitive medical data. Patient informed consent does not include agreement for publicly sharing acquired image data.
